# Association of aggregate index of systemic inflammation with increased all-cause and cardiovascular mortality in female cancer patients

**DOI:** 10.3389/fonc.2025.1552341

**Published:** 2025-04-29

**Authors:** Ying Yang, Zelin Hu, Yuqin Ye, Haoqi Wu, Wei Sun, Ning Wang

**Affiliations:** ^1^ Dalian Medical University, Dalian, China; ^2^ The Dandong Central Hospital, Dandong, China; ^3^ The Second Hospital of Dalian Medical University, Dalian, China; ^4^ Medical College of Wuhan University of Science and Technology, Wuhan, China

**Keywords:** female cancer, aggregate index of systemic inflammation, AISI, mortality, NHANES

## Abstract

**Background:**

Cancer is a leading cause of death, especially among women, with cancers like breast, ovarian, and cervical cancer presenting unique diagnostic and treatment challenges. Systemic inflammation plays a significant role in cancer progression, affecting both tumor development and therapeutic outcomes. Despite the established link between inflammation and cancer, comprehensive studies on the prognostic value of the Aggregate Index of Systemic Inflammation (AISI) in female cancer patients are lacking. This study explores the association between AISI and mortality outcomes, including all-cause and cardiovascular mortality, in female cancer patients.

**Methods:**

This study analyzes data from the NHANES database and Dandong Central Hospital. Kaplan-Meier survival curves and multivariable Cox proportional hazards regression analyses were used to assess the relationship between AISI and all-cause and cardiovascular mortality. Restricted cubic spline plots and subgroup analyses were applied to explore potential interactions.

**Results:**

Elevated AISI levels were strongly associated with increased all-cause and cardiovascular mortality. Patients in the highest AISI quartile demonstrated significantly higher mortality risks compared to those in the lowest quartile. ROC curve analysis indicated superior predictive performance of AISI over SII. Restricted cubic spline plots revealed a linear relationship, with mortality risk notably increasing when AISI levels were elevated.

**Conclusion:**

AISI is a robust predictor of all-cause and cardiovascular mortality in female cancer patients. Its ease of measurement and strong prognostic value make it a valuable tool for risk assessment and management in this population.

## Introduction

In 2022, nearly 20 million new cancer cases were reported, alongside 9.7 million cancer-related deaths. It is estimated that approximately one in five individuals, regardless of gender, will develop cancer in their lifetime, while roughly one in nine men and one in twelve women are expected to succumb to the disease (1). A recent study highlighted the disproportionate cancer mortality among women, revealing that approximately one million children lost their mothers to cancer in 2020. Nearly half of these maternal deaths were attributed to breast or cervical cancer (2). Among female patients, certain cancers, such as breast, ovarian, and cervical cancers, have higher rates of occurrence. These types of cancer are closely linked to women’s physiological characteristics and hormonal environments, which makes their diagnosis and treatment more complex (3). Therefore, improving cancer treatment strategies to better meet the needs of female patients can reduce the risk of complications and enhance long-term survival outcomes (4, 5).

In recent years, growing evidence has shown that systemic inflammatory responses play a critical role in the onset and progression of cancer (6, 7). Inflammation is involved in various stages of tumor development, including initiation, promotion, malignant transformation, invasion, and metastasis. Furthermore, inflammation has a significant impact on immune surveillance and therapeutic responses (8). AISI has been widely used to explore the link between inflammation and various diseases (9–11). As a systemic inflammation indicator, AISI has emerged as a practical and valuable tool. This biomarker is characterized by ease of collection, rapid results, low cost, and high efficiency and reliability, making it accessible through routine blood tests, offering notable advantages for clinical application. Additionally, the Systemic Immune-Inflammation Index (SII), another immunoinflammatory marker, has been used to predict and evaluate the prognosis of various solid tumors, including gastric cancer, small-cell lung cancer, and ovarian cancer (12–15). However, while AISI has been applied in studies of idiopathic pulmonary fibrosis (IPF), COVID-19, hypertension, and certain cancers (16–19), research on its prognostic value in female cancer patients remains limited.

Against this background, the present study aims to investigate the potential association between AISI, a blood-based inflammatory biomarker, and mortality outcomes (including all-cause mortality and cardiovascular events) in female cancer patients. Additionally, the study seeks to evaluate the comparative advantages of AISI and SII in prognostic assessments, providing clinicians with a novel, convenient, and reliable tool for risk assessment and management strategies for female cancer patients.

The National Health and Nutrition Examination Survey (NHANES) is a nationally representative cross-sectional survey aimed at assessing the health and nutritional status of the U.S. population. Utilizing a large, nationally representative dataset from the NHANES database, this study examines the association between the novel inflammatory biomarker AISI and all-cause as well as cardiovascular mortality in female cancer patients.

## Methods

### Data source and study population

**Figure 1:**
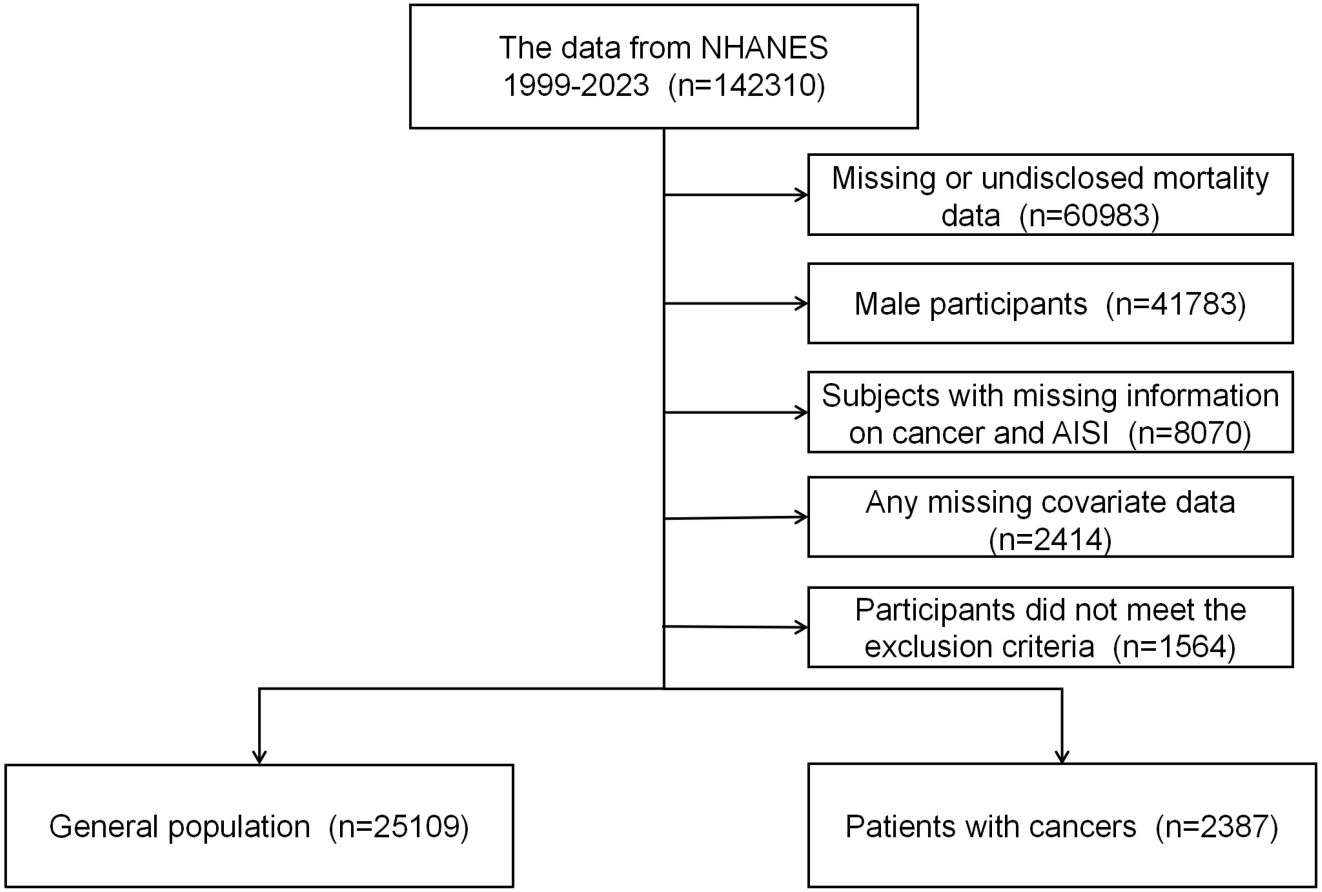
Selection process for study cohorts.

This study utilized two datasets: one derived from the NHANES database and the other from inpatient data of Dandong Central Hospital. Sample 1 comprises participants from the NHANES database, covering the period from 1999 to 2023. NHANES is organized and administered by the National Center for Health Statistics (NCHS), utilizing a nationally representative, stratified, multistage probability sampling method (20). The database is managed and maintained by NCHS, with all participants providing written informed consent, and the study received approval from the NCHS Institutional Review Board (IRB). Additional information is available at: http://www.cdc.gov/nchs/nhanes/irba98.htm. Given that NHANES is a publicly available dataset containing anonymous data, this study did not require further ethical approval or informed consent. The study adhered to ethical guidelines for the protection of human subjects’ safety and privacy as outlined by relevant authorities and data administrators. The study cohort comprised adult female participants diagnosed with cancer, along with a general population sample. The exclusion criteria were as follows: 1) All male participants and female individuals under the age of 18; 2) participants with missing hematologic laboratory test data on neutrophils (NEU), platelets (PLT), monocytes (MON), or lymphocytes (LYM); 3) individuals lacking covariate data; 4) individuals with incomplete or undisclosed mortality data; and 5) participants without available sample weight data. In total, 2,387 participants were included in the analysis. The participant selection process is depicted in **Figure 1**.

Sample 2 includes electronic medical record data from female patients diagnosed with cancer at our hospital between January 2022 and December 2023. The dataset records baseline characteristics and laboratory test results at admission. Blood samples were collected, processed, and analyzed following standardized international biochemical laboratory protocols. This study was conducted in accordance with the ethical guidelines outlined in the 1964 Declaration of Helsinki and received approval from the IRB; therefore, written informed consent was not required. The inclusion criteria were as follows: (1) absence of a pathological report confirming cancer diagnosis; (2) incomplete laboratory test or electronic medical record data; (3) presence of underlying diseases that directly affect four key hematological parameters, such as infections, liver cirrhosis, exogenous albumin supplementation, or leukemia; (4) lack of complete follow-up records. Ultimately, 116 participants were included, with the detailed screening process illustrated in **Supplementary Figure 1**.

### Cancer status and mortality

The diagnosis of cancer or malignant neoplasm was based on responses to the following questions from the NHANES questionnaire (MCQ220, MCQ230A, MCQ230B, MCQ230C): 1) Has a doctor or other healthcare professional ever told you that you had cancer or a malignant tumor? 2) If yes, what type of cancer was it?

NHANES data were linked with mortality data from the National Death Index (NDI) through December 31, 2019, which is publicly available. This linkage facilitated the determination of mortality outcomes in the study population. To ensure accurate data matching, a probabilistic matching algorithm was applied. Causes of death were classified according to the International Classification of Diseases, 10th Revision (ICD-10) (21). With the NCHS classifying heart diseases (054–064) and all other causes (010) for our study (21).

### AISI definition

AISI was derived using Beckman Coulter’s counting and classification method, based on parameters from a complete blood count (CBC). This method involves automated sample handling, including dilution and mixing, with hemoglobin measurements conducted via a single-beam photometer. White blood cell (WBC) classification was performed using VCS technology. CBC analysis of blood samples was conducted at the NHANES mobile examination center (MEC) using the Beckman Coulter DxH 800 device, which also provided blood cell distribution data. According to previous studies, the AISI formula is calculated as (NEU * PLT * MONO)/LYM (22). Additionally, due to the left-skewed distribution of inflammatory markers observed in regression analysis, a logarithmic (ln) transformation of the AISI was applied.

### Covariates

This study identified independent risk factors associated with female cancer and incorporated them as covariates in the analysis. These covariates were selected based on prior research to minimize potential confounding bias. Specifically, the included covariates were age, race, education level, income-to-poverty ratio, body mass index (BMI), smoking status, alcohol consumption, diabetes, and hypertension. Demographic information, including age, race, education level, and income-to-poverty ratio, was collected by trained interviewers using the computer-assisted personal interview (CAPI) system. Anthropometric measurements were obtained by trained health technicians at the NHANES MEC, and BMI was calculated as weight (kg) divided by height (m) squared.

Smoking status was categorized based on participants’ responses to two survey questions: “Have you ever smoked 100 or more cigarettes?” and “Do you currently smoke?” Participants were classified into three groups: never smokers, former smokers, and current smokers. Never smokers were defined as individuals who had never smoked 100 or more cigarettes and were not currently smoking. Current smokers were those who had smoked 100 or more cigarettes and were still smoking at the time of the survey, while former smokers were those who had smoked 100 or more cigarettes in the past but had since quit. Alcohol consumption was categorized according to self-reported drinking frequency into four groups: heavy drinkers, moderate drinkers, light drinkers, and non-drinkers. Heavy drinkers were defined as individuals consuming four or more alcoholic beverages per day, moderate drinkers as those consuming up to three drinks per day, light drinkers as those who had consumed alcohol fewer than 12 times in the past year, and non-drinkers as those who reported never consuming alcohol.

Diabetes and hypertension diagnoses were determined using a combination of questionnaire responses and laboratory data to enhance diagnostic accuracy. The diabetes-related questionnaire included questions such as: “Has a doctor ever diagnosed you with diabetes?” “Do you take insulin?” and “Do you take oral hypoglycemic medications?” Laboratory diagnostic criteria for diabetes were defined as fasting blood glucose levels ≥7.0 mmol/L, HbA1c ≥6.5%, or oral glucose tolerance test (OGTT) blood glucose levels ≥11.1 mmol/L. Similarly, hypertension was diagnosed based on multiple blood pressure measurements ≥130/80 mmHg or a self-reported physician diagnosis.

### Statistical analysis

To ensure the sample**’**s national representativeness, MEC weights from the NHANES sampling design were applied following the NHANES weighting guidelines. Continuous variables were reported as weighted means with their corresponding standard errors, while categorical variables were expressed as frequencies and weighted proportions. The chi-square test was employed to evaluate differences in categorical variables, and the Kruskal-Wallis test was utilized to compare continuous variables across AISI quartiles (Q1–Q4).

Multivariable Cox proportional hazards regression analysis was conducted to investigate the relationship between AISI and mortality, with adjustments made for demographic characteristics (Model 2) and all covariates (Model 3). Hazard ratios (HRs) and 95% confidence intervals (CIs) were calculated to quantify the strength of these associations. Kaplan-Meier survival curves were constructed to visualize survival probabilities across different AISI quartiles, and statistical significance of the differences was assessed.

Additionally, we generated the receiver operating characteristic (ROC) curves for AISI and SII in relation to both all-cause and cardiovascular mortality rates to assess their predictive performance in female cancer patients. To further explore these relationships, we employed restricted cubic spline methods to visually demonstrate the potential linear association between AISI and both all-cause and cardiovascular mortality rates. For a more in-depth analysis, we conducted threshold analysis to examine the association between AISI and mortality rates. Subgroup analysis was performed to assess the potential impact of other variables on these relationships, thereby validating the robustness of our findings. Finally, we implemented mediation analysis to investigate the mediating effect of AISI on the relationship between cancer and both all-cause and cardiovascular mortality in female patients.

All statistical tests were two-sided, with a p-value of less than 0.05 considered indicative of statistical significance. Data analysis was performed using IBM SPSS Statistics version 25.0 and R version 4.3.1.

## Results

### Study population and baseline characteristics

Between 1999 and 2023, a total of 142,310 participants from the NHANES database were included in this study. After excluding individuals who did not meet the study criteria or had incomplete data, the final cohort comprised 2,387 female cancer patients and 25,109 individuals from the general population (**Figure 1**). The mean age of participants was 62 ± 15 years. Baseline characteristics are summarized in **Table 1**. Significant differences were observed across groups in terms of age, race, education level, hypertension, and diabetes according to the quartiles of AISI (p < 0.05). **Supplementary Table 1** presents the clinical characteristics of the patients in Sample 2. The study population is predominantly concentrated around the age of 60.

**Table 1:** Baseline characteristics of the study cohort.

Study variables		Total (n=2387)	Quartiles of AISI				P value
			Q1: <5.17 (n = 597)	Q2: 5.17-5.60 (n = 595)	Q3: 5.60-6.04 (n = 600)	Q4: >6.04 (n = 595)	
Age, years		62.75 ± 15.74	60.88 ± 14.95	61.33 ± 15.58	64.19 ± 15.86	64.59 ± 16.23	<0.001
Race							<0.001
Mexican		221 (9.26%)	60 (10.05%)	60 (10.08%)	51 (8.50%)	50 (8.40%)	
Hispanic		142 (5.95%)	41 (6.87%)	36 (6.05%)	32 (5.33%)	33 (5.55%)	
Non-Hispanic white		1623 (67.99%)	351 (58.79%)	412 (69.24%)	423 (70.50%)	437 (73.45%)	
Non-Hispanic black		282 (11.81%)	110 (18.43%)	59 (9.92%)	65 (10.83%)	48 (8.07%)	
Other/multiracial		119 (4.99%)	35 (5.86%)	28 (4.71%)	29 (4.83%)	27 (4.54%)	
Education level, n (%)							0.006
Never attended high school		638 (26.73%)	190 (31.83%)	155 (26.05%)	156 (26.00%)	137 (23.03%)	
High school and above		1749 (73.27%)	407 (68.17%)	440 (73.95%)	444 (74.00%)	458 (76.97%)	
Poverty-to-income ratio, n (%)							0.906
Poor (≤1)		459 (19.23%)	121 (20.27%)	112 (18.82%)	113 (18.83%)	113 (18.99%)	
Not poor (>1)		1928 (80.77%)	476 (79.73%)	483 (81.18%)	487 (81.17%)	482 (81.01%)	
Smoking status, n (%)							0.572
Never		138 (5.78%)	35 (5.86%)	37 (6.22%)	36 (6.00%)	30 (5.04%)	
Former		1487 (62.30%)	376 (62.98%)	357 (60.00%)	389 (64.83%)	365 (61.34%)	
Current smoker		762 (31.92%)	186 (31.16%)	201 (33.78%)	175 (29.17%)	200 (33.61%)	
Alcohol use, n (%)							0.065
Never		479 (20.07%)	101 (16.92%)	123 (20.67%)	120 (20.00%)	135 (22.69%)	
Mild		411 (17.22%)	105 (17.59%)	93 (15.63%)	107 (17.83%)	106 (17.82%)	
Moderate		1350 (56.56%)	365 (61.14%)	337 (56.64%)	338 (56.33%)	310 (52.10%)	
Heavy		147 (6.16%)	26 (4.36%)	42 (7.06%)	35 (5.83%)	44 (7.39%)	
Hypertension, n (%)		1273 (53.33%)	286 (47.91%)	305 (51.26%)	330 (55.00%)	352 (59.16%)	<0.001
Diabetes mellitus, n (%)		446 (18.68%)	107 (17.92%)	95 (15.97%)	107 (17.83%)	137 (23.03%)	0.013
BMI, kg/m2		29.22 ± 6.80	29.08 ± 6.76	28.77 ± 6.17	29.34 ± 6.72	29.68 ± 7.46	0.122
Laboratory tests							
Neutrophil count, 109/L		4.36 ± 1.79	3.08 ± 1.65	3.89 ± 1.07	4.52 ± 1.17	5.95 ± 1.78	<0.001
Lymphocyte count, 109/L		2.30 ± 7.53	3.16 ± 14.97	2.12 ± 0.74	2.04 ± 0.77	1.87 ± 0.76	0.014
monocytes count, 109/L		0.56 ± 0.22	0.44 ± 0.24	0.51 ± 0.13	0.58 ± 0.15	0.70 ± 0.25	<0.001
Platelet count, 109/L		255.35 ± 69.83	214.80 ± 58.30	245.63 ± 57.45	264.22 ± 61.70	296.81 ± 74.07	<0.001

### Mortality outcomes

**Figure 2:**
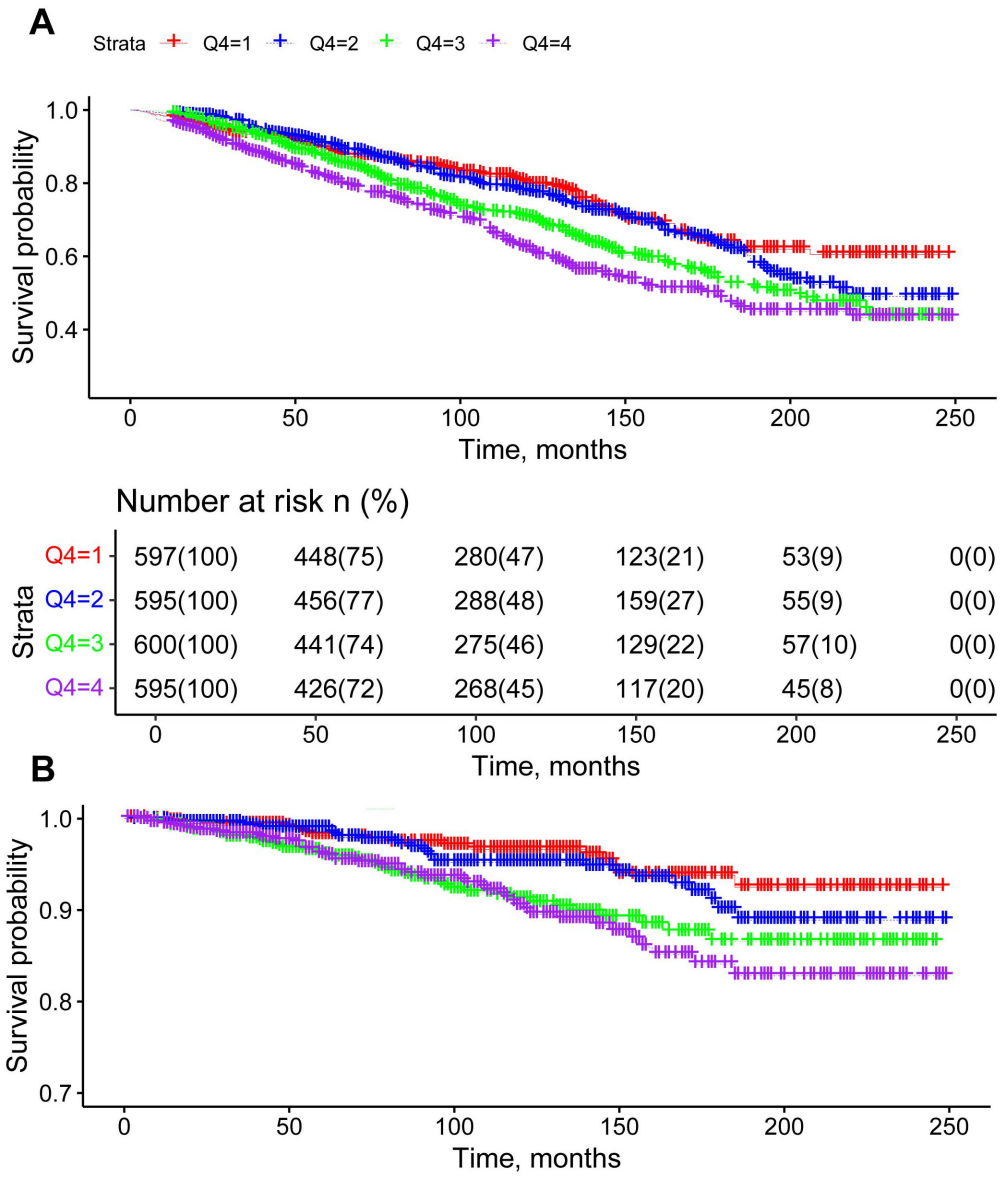
Kaplan-Meier survival curves for all-cause mortality **(A)** and cardiovascular mortality **(B)** stratified by AISI quartiles, adjusted for age, race, education, poverty-to-income ratio, hypertension, diabetes, BMI, alcohol use, and smoking.

During the 20-year follow-up period, Kaplan-Meier survival curves indicated 678 cases of all-cause mortality and 136 cases of cardiovascular mortality. Higher quartiles of AISI were associated with reduced survival rates, highlighting an independent association between elevated AISI levels and increased all-cause and cardiovascular mortality among adult female cancer patients in the United States (**Figure 2**).

### Comparison of predictive performance: AISI vs. SII

**Figure 3:**
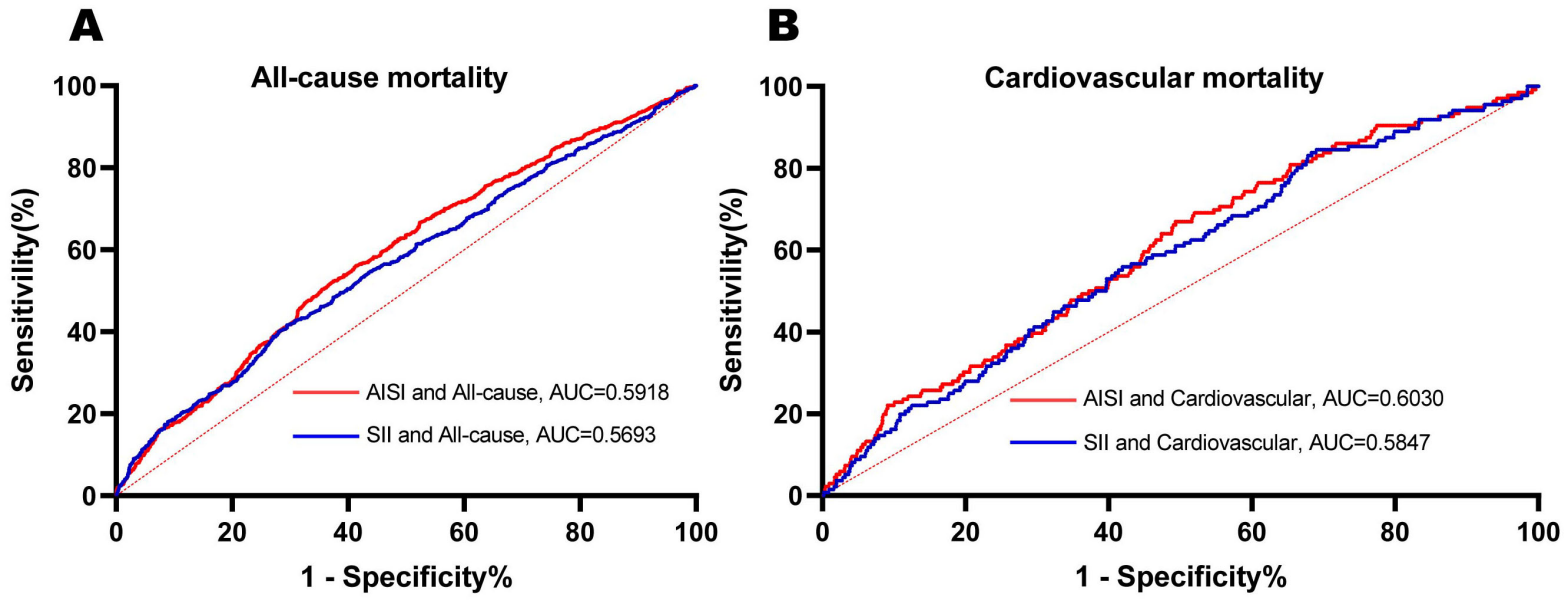
ROC curves were utilized to assess the differences in predictive abilities of SII and AISI for all-cause **(A)** and cardiovascular **(B)** mortality in female cancer patients. ROC receiver operating characteristic curve, AUC area under the curve.

Furthermore, ROC curves were employed to compare the predictive performance of SII and AISI in female cancer patients. As shown in **Figure 3**, AISI demonstrated superior predictive ability for both all-cause and cardiovascular mortality. Specifically, the area under the curve (AUC) for AISI predicting all-cause mortality was 0.5918, while the AUC for SII was slightly lower at 0.5693. Similarly, for cardiovascular mortality, AISI showed a higher AUC of 0.6030 compared to SII’s AUC of 0.5847. In addition, we calculated the Net Reclassification Improvement (NRI) and Integrated Discrimination Improvement (IDI) to further evaluate the improvement in classification and risk stratification with AISI compared to SII. As shown in **Supplementary Table 2**, the NRI and IDI analyses confirmed that AISI provided a significantly better reclassification of patients’ mortality risks, particularly in identifying high-risk individuals. The Harrell’s Concordance Index also indicated a higher concordance between AISI’s predictions and actual mortality events, further supporting its superior predictive ability.

### Regression analysis of AISI and mortality risk

Proportional hazards regression analysis identified a positive correlation between AISI levels and mortality. This association remained robust after adjusting for various covariates across Models 2 and 3. Specifically, each one-unit increase in AISI was associated with a 35% increase in the risk of all-cause mortality (95% CI: 1.20–1.51) and a 48% increase in the risk of cardiovascular mortality (95% CI: 1.14–1.94). Additionally, the data from Sample 2 yielded similar findings. Cox regression analysis was performed on the Sample 2 population, and the results further demonstrated a positive association between AISI and all-cause mortality in female cancer patients (1.57 [1.12, 2.21]). For detailed results, please refer to **Supplementary Table 3**. Compared to patients in the lowest AISI quartile (Q1), those in the highest quartile (Q4) exhibited significantly higher risks for both all-cause and cardiovascular mortality. Furthermore, patients in the third AISI quartile (Q3) demonstrated an elevated risk of cardiovascular mortality relative to those in Q1. These findings underscore a strong association between higher AISI quartiles and increased risks of all-cause and cardiovascular mortality. AISI may serve as a valuable indicator for assessing patient mortality risk, particularly for cardiovascular events. The independent positive correlation between AISI, analyzed as both a continuous and categorical variable, and mortality is further supported by detailed effect sizes presented in **Table 2**.

**Table 2:** HRs (95% CIs) for all-cause and cardiovascular mortality according to different types of AISI data in cancer patients from NHANES (1999–2023) among U.S. Adult females.

AISI		Model1*			Model2*			Model3*	
		HR (95% CI)	p value		HR (95% CI)	p value		HR (95% CI)	p value
All-cause mortality									
Continuous data		1.50(1.34,1.69)	<0.0001		1.37(1.22,1.54)	<0.0001		1.35(1.20,1.51)	<0.0001
Quartiles	Q1	Reference			Reference			Reference	
	Q2	1.11(0.87,1.41)	0.3907		1.06(0.84,1.35)	0.6231		0.95(0.75,1.21)	0.6851
	Q3	1.46(1.17,1.83)	0.001		1.24(0.98, 1.56)	0.0689		1.21(0.96,1.53)	0.1025
	Q4	1.82(1.46,2.27)	<0.0001		1.61(1.29,2.02)	<0.0001		1.52(1.22,1.91)	0.0003
Cardiovascular mortality									
Continuous data		1.81(1.39,2.35)	<0.0001		1.56(1.20,2.03)	0.0008		1.48(1.14,1.94)	0.0035
Quartiles	Q1	Reference			Reference			Reference	
	Q2	1.35(0.75,2.42)	0.3188		1.24(0.69,2.24)	0.4716		1.05(0.58,1.91)	0.8774
	Q3	2.29(1.34,3.93)	0.0025		1.85(1.07,3.20)	0.0268		1.74(1.01,3.01)	0.0477
	Q4	2.55(1.49,4.35)	0.0006		2.12(1.23,3.63)	0.0066		1.84(1.06,3.19)	0.0295

CI, Confidence Interval; HR, Hazard Ratio; AISI, The aggregate index of systemic inflammation.

*Model 1, non-adjusted. Model 2, adjusted for age, education, poverty-to-income ratio and race. Model 3, adjusted for age, race, education, poverty-to-income ratio, hypertension, diabetes, alcohol use, smoking and BMI.

### Linear relationship between AISI and mortality

**Figure 4:**
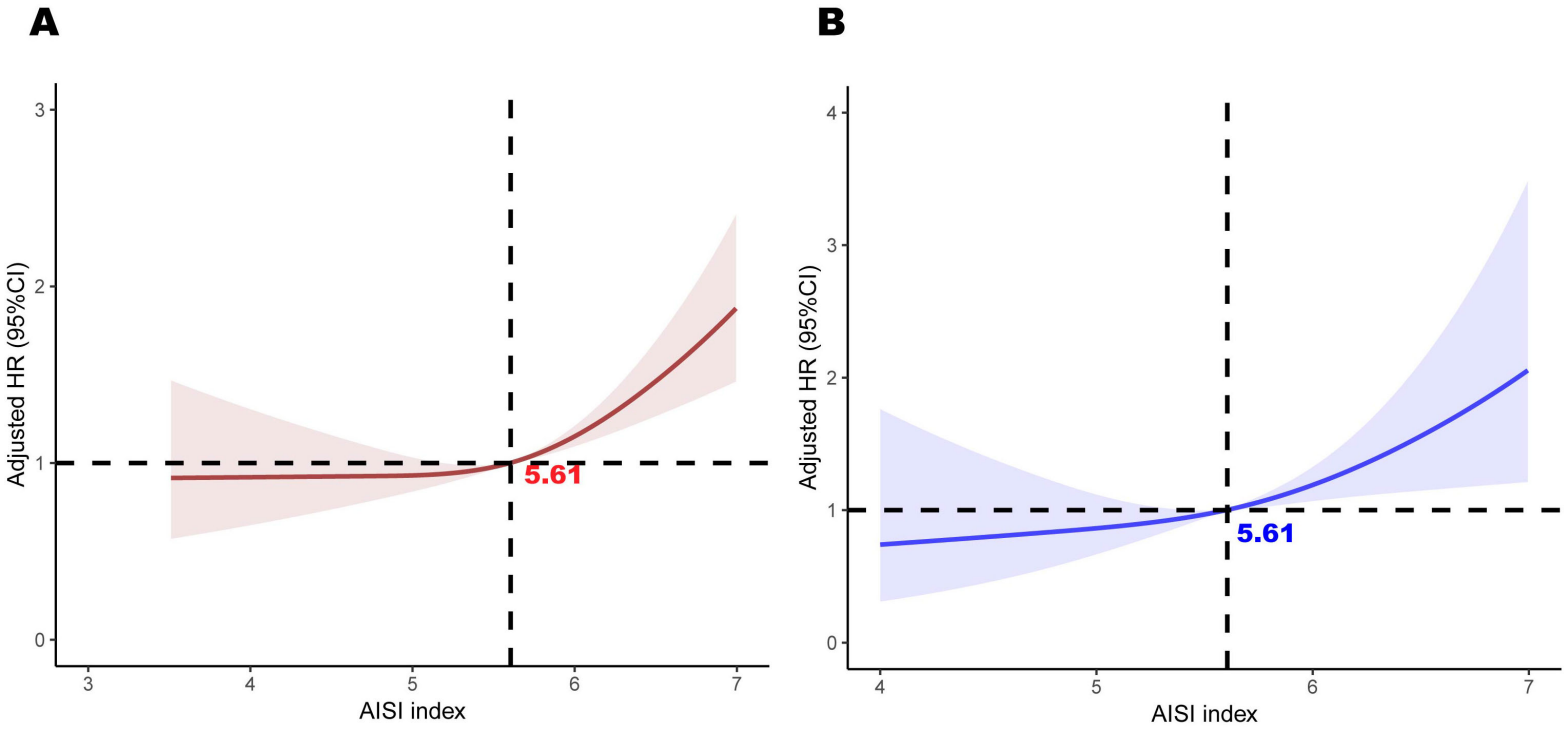
Association between AISI and all-cause mortality **(A)** and cardiovascular mortality **(B)** in participants with female cancer, adjusted for age, race, education, poverty-to-income ratio, hypertension, diabetes, BMI, alcohol use, and smoking. The shaded areas represent the 95% CI.

Restricted cubic spline plots revealed a clear linear relationship between AISI and both all-cause and cardiovascular mortality (linear P < 0.05, **Figure 4**). This analysis was adjusted for all covariates. Notably, when AISI reached a value of 5.61, the hazard ratio (HR) for both all-cause and cardiovascular mortality was 1, indicating a threshold at which the risk of mortality transitions from low to high. AISI levels exceeding this threshold were significantly associated with a poorer prognosis among female cancer patients. We also applied RCS curves to the Sample 2 population to visualize the results and determined that an AISI level of 5.6 resulted in an AISI risk ratio equal to 1. (See **Supplementary Figure 2** for details).

### Subgroup analysis

**Figure 5:**
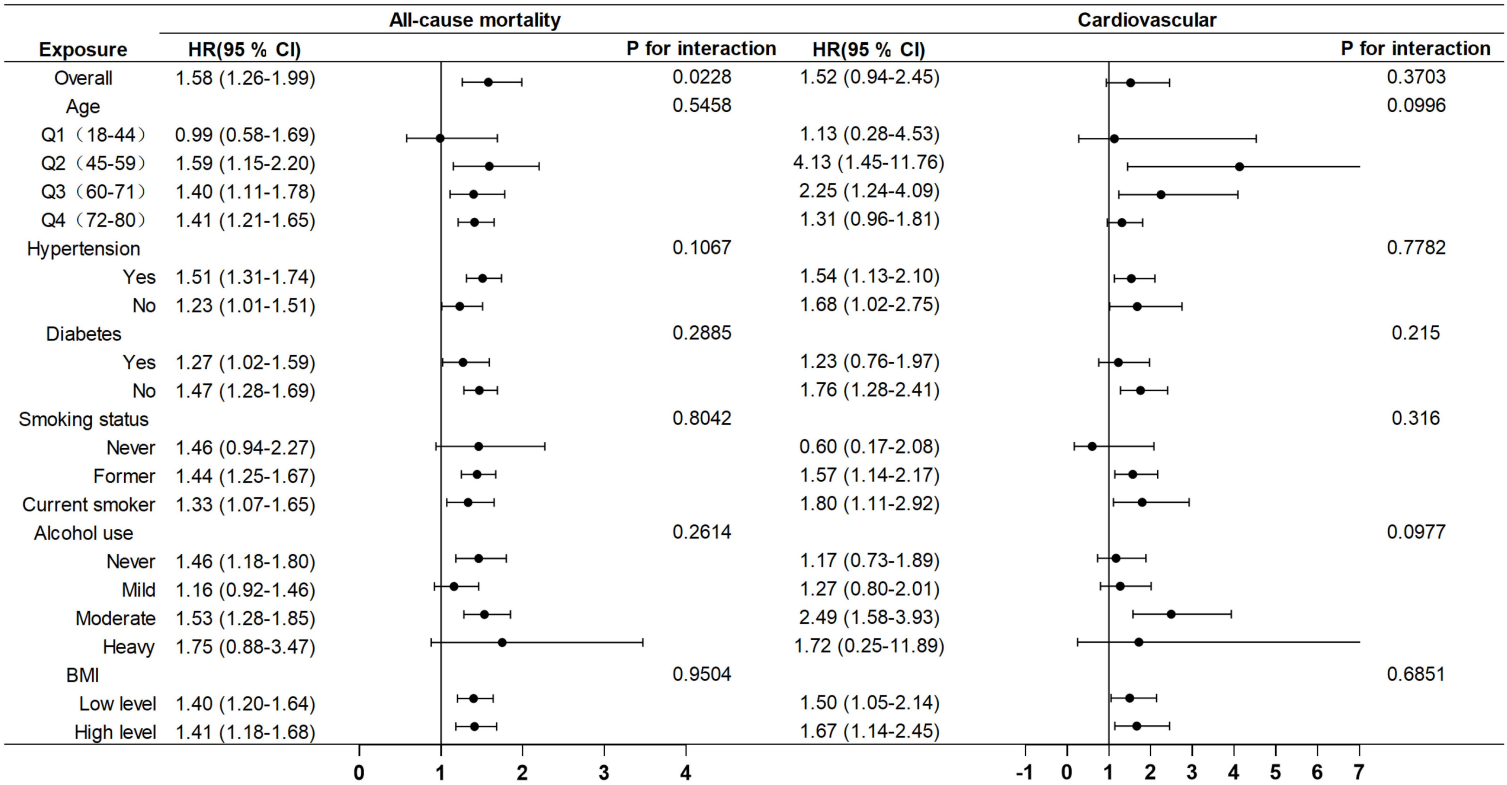
Subgroup analysis of the associations between AISI and all-cause and cardiovascular mortality, adjusted for age, race, education, poverty-to-income ratio, hypertension, diabetes, BMI, alcohol use, and smoking.

Subgroup analyses were conducted to evaluate the influence of other covariates on the association between AISI and mortality (**Figure 5**). Hazard ratio (HR) of 1 based on Cox proportional hazards regression analysis was used as the reference point for the threshold. By categorizing AISI into low- and high-risk groups based on the threshold associated with the lowest risk, the analysis revealed that female cancer patients with elevated AISI levels (AISI > 5.61) exhibited a significantly higher risk of all-cause mortality compared to those with lower AISI levels (HR = 1.58, 95% CI: 1.26–1.99). These findings suggest that elevated AISI levels serve as a strong predictor of increased mortality risk.

### Mediation analysis

Finally, the results of the mediation analysis showed that AISI did not play a mediating role in the effect of cancer on all-cause mortality and cardiovascular mortality in female patients (**Table 3**).

**Table 3:** Mediation analysis of the association between Female cancer patients and the risk of all cause and cardiovascular mortality mediated by AISI.

	Non-adjusted β (95%CI)P-value	Adjust II β(95%CI) P-value
AISI		
All-cause mortality		
Direct effect	0.171 (0.154, 0.190) <0.0001	0.013 (0.004, 0.023) 0.002
Indirect effect	0.002 (0.001, 0.003) 0.002	0.001 (-0.0002, 0.002) 0.104
Total effect	0.173 (0.156, 0.192) <0.0001	0.014 (0.005, 0.024) 0.002
PM, %	1.2	5
P-value	0.002	0.106
Cardiovascular mortality		
Direct effect	0.027 (0.018, 0.037) <0.0001	-0.008 (-0.013, -0.004) <0.0001
Indirect effect	0.001 (0.0001, 0.001) 0.002	0.0002 (-0.00004, 0.0003) 0.104
Total effect	0.028 (0.018, 0.038) <0.0001	-0.008 (-0.013, -0.003) <0.0001
PM, %	2.2	-2
P-value	0.002	0.104

Crude model: we did not adjust other covariant.

Model II on AISI: we adjusted age, race, education, poverty-to-income ratio, hypertension, diabetes, alcohol use, smoking and BMI.

## Discussion

In this retrospective cohort study, we employed two datasets to investigate the association between AISI and both all-cause and cardiovascular mortality in female cancer patients. The study’s primary finding revealed a robust, positive correlation between AISI—analyzed both as a continuous variable and categorized into quartiles—and mortality outcomes in this population. This relationship persisted even after adjusting for a range of potential confounding factors. Notably, when compared with the previously established SII, AISI exhibited superior predictive accuracy for mortality risk in female cancer patients. Subgroup analyses further underscored that elevated AISI levels were strongly linked to a heightened risk of all-cause mortality. However, in the studied cohort, covariates such as age, hypertension, diabetes, smoking, alcohol consumption, and body mass index did not significantly influence the relationship between AISI and mortality (all P-values > 0.05). Moreover, no significant interactions were observed between AISI and these covariates, indicating that AISI may serve as a robust independent predictor of mortality in this patient population, with minimal confounding from other established risk factors. By addressing a critical research gap, our findings highlight the potential utility of AISI as a valuable prognostic marker for assessing disease severity and clinical outcomes in female cancer patients.

Inflammation significantly contributes to cancer development and progression through multiple biological pathways. Growing evidence underscores a strong association between inflammation and cancer progression, as demonstrated in recent studies (23–26). In the context of female cancers, chronic inflammation and sustained immune activation have been identified as critical factors driving disease progression (27). AISI, a composite biomarker of systemic inflammation, is constructed using key inflammatory components, including neutrophils, platelets, monocytes, and lymphocytes.

Inflammation and immune cells within the tumor microenvironment (TME) play a pivotal role in influencing the growth and progression of cancer cells (28, 29). Among these, neutrophils are the most abundant immune cells in the TME and have a dual role in tumor biology. Elevated neutrophil counts suppress the secretion of tumor necrosis factor-α, which subsequently increases circulating levels of vascular endothelial growth factor (VEGF). The overexpression of VEGF facilitates tumor angiogenesis, thereby accelerating tumor growth and metastasis (30). Moreover, neutrophils secrete various inflammatory mediators, including matrix metalloproteinase-9, neutrophil elastase (NE), and interleukin-8, all of which contribute to tumor proliferation and metastasis (31, 32). Conversely, neutrophils also exhibit antitumor properties by releasing neutrophil extracellular traps (NETs), which mediate tumor cell destruction through components such as histones, NE, and myeloperoxidase (MPO) (33–35). Richardson et al. further demonstrated a correlation between NETs released by activated neutrophils and poor prognosis in patients with colorectal cancer (36).

Neutrophilia is often accompanied by relative lymphocytopenia, highlighting a potential weakening of cell-mediated adaptive immune responses (37). Lymphocytes, as key mediators of immune surveillance and immunoediting, play a critical role in antitumor immunity, with their infiltration into TME serving as a fundamental requirement for effective immune responses against tumors (38). Tumor-infiltrating lymphocytes are strongly associated with improved survival across various cancers, whereas low lymphocyte counts or inadequate infiltration are linked to poorer survival outcomes (39). Specifically, lymphocytopenia has been identified as an independent prognostic factor for both overall survival and progression-free survival in patients with metastatic breast cancer (40). Moreover, interactions between CD8+ cytotoxic T lymphocytes and CD4+ helper T lymphocytes are essential for inducing tumor cell apoptosis via immune-mediated antitumor mechanisms (41). Overall, diminished lymphocyte counts may reflect weak or insufficient tumor-specific immune responses, underscoring the critical importance of lymphocytes in cancer immunology.

Monocytes are widely recognized as protumorigenic cells, serving as major sources of chemokines and cytokines within TME, thereby driving tumor progression and metastasis (42, 43). Tumor-secreted CCL5 and CXCL8 also play roles in recruiting monocytes, neutrophils, and other leukocytes, which can differentiate into TAMs and tumor-associated neutrophils (TANs), both of which can assume pro-tumorigenic roles (44). Furthermore, tumor cells can drive the substantial accumulation of tumor-promoting myeloid immune cells within the tumor microenvironment. For instance, myeloid-derived suppressor cells (MDSCs), tumor-associated macrophages (TAMs), and regulatory T cells have been identified as the predominant tumor-promoting immune cells in the tumor microenvironment (45). Studies, such as those conducted by Bingle et al. (46), have demonstrated that macrophage density is strongly associated with poor clinical prognosis in various solid tumors. Additionally, research has revealed that TAMs promote tumor cell migration, invasion, and angiogenesis while simultaneously suppressing antitumor immune responses, thus facilitating tumor dissemination and metastasis (47, 48). Consistent with these findings, elevated peripheral monocyte levels have been correlated with poor prognoses across multiple cancer types (49–51).

Platelets, beyond their conventional roles in hemostasis and wound healing, are actively involved in all stages of tumor progression. Recent studies highlight the critical role of platelets within TME. Platelets interact with TME components via membrane proteins and secrete cytokines that regulate tumor growth, metastasis, and invasion (52). During hematogenous metastasis, platelets shield tumor cells, enhance their survival, and facilitate immune evasion, thereby promoting ectopic metastasis (53). Additionally, the interaction between tumor cells and platelets triggers platelet activation, elevates platelet counts, and increases the risk of thrombosis in cancer patients (54). Elevated platelet counts have been identified as adverse prognostic indicators across various cancer types (54, 55). Furthermore, Nicholas et al. (56)demonstrated that the heightened cardiovascular risk observed in female cancer patients is influenced by multiple factors, including chronic inflammation, oxidative stress, metabolic dysregulation, clonal hematopoiesis, gut dysbiosis, hormonal alterations, and cellular senescence. These exacerbated immune-inflammatory responses not only accelerate cancer progression but also contribute to irreversible cardiovascular damage, significantly increasing mortality and the incidence of adverse cardiovascular events (57). Thus, mitigating the overactivation of immune-inflammatory pathways may help delay cancer progression and reduce associated cardiovascular risks.

Our study presents several key strengths. First, the cohort analysis is based on 15 cycles of the US NHANES from 1999 to 2023, which utilizes a rich dataset and spans a long survey period, thereby ensuring the robustness and reliability of the results. Second, to further validate the research findings, we utilized clinical data from Dandong Central Hospital as a validation set, thereby enhancing the external validity of the results and improving the generalizability and applicability of the conclusions. Third, Numerous studies have demonstrated that conventional inflammatory markers, such as the neutrophil-lymphocyte ratio (NLR), platelet-lymphocyte ratio (PLR), and C-reactive protein (CRP), possess strong prognostic predictive capabilities in cancer patients (58–63). However, AISI is considered to offer a more comprehensive reflection of the systemic inflammatory status compared to traditional markers, particularly excelling in the prediction of poor prognosis. A study involving patients with IPF found that AISI demonstrated the highest prognostic value among various inflammatory markers, significantly outperforming NLR and PLR (19). This advantage is primarily attributed to AISI’s incorporation of monocyte count, which is more sensitive to inflammatory responses, thereby providing a more accurate assessment of the systemic inflammatory state. Finally, our study is the first to systematically investigate the relationship between the inflammation biomarker AISI and both all-cause and cardiovascular mortality in female cancer patients. Our aim is to identify novel prognostic indicators for the elevated mortality risk in this population, providing clinicians with a new tool for identifying high-risk individuals. Not only that, but we employed multiple analytical approaches, including Cox proportional hazards regression, subgroup analysis, and Kaplan-Meier survival curves, all of which further substantiate the effectiveness of AISI as a reliable predictor of mortality risk in female cancer survivors.

## Limitations

While our study provides significant insights, it is important to recognize its limitations. First, the use of cross-sectional laboratory data restricts our ability to accurately assess longitudinal changes or responses to interventions. To address this limitation, dynamic monitoring of AISI levels could offer a more comprehensive understanding of temporal fluctuations in immune-inflammatory status. Second, we lacked access to detailed clinical information on cancer patients, including TNM staging and tumor size. Although tumor stratification was performed, and confounding factors were controlled, residual confounding by unmeasured variables remains a possibility. Factors such as treatment regimens and genetic variations, which were not included in the NHANES dataset, may influence the stability of our results. Future clinical cohort studies are necessary to validate our findings and examine the potential impact of these variables. Third, the reliance on household interviews and questionnaires in the NHANES dataset introduces risks of reporting inaccuracies and recall bias. Although rigorous validation processes are employed by agencies like the NCHS, these inherent limitations cannot be completely eliminated.

## Conclusions

By investigating the characteristics of the inflammatory biomarkers AISI in female cancer patients, we found that AISI is linearly positively correlated with both all-cause mortality and cardiovascular mortality in this population. AISI is not only a valuable prognostic biomarker, but its simplicity, ease of measurement, and cost-effectiveness also underscore its broad potential for clinical application. High-risk patients, as determined by AISI levels, can benefit from enhanced cardiovascular disease monitoring and intervention, which may ultimately reduce mortality risk. It is recommended that AISI be incorporated into the routine screening process for female cancer patients. Furthermore, when combined with other clinical indicators, AISI can contribute to the development of personalized health management strategies. For instance, if AISI exceeds 5.61, intensified cardiovascular disease interventions should be considered, and cancer treatment regimens may be adjusted accordingly. Therefore, our study highlights the potential clinical value of the inflammation biomarker AISI in predicting the prognosis of female cancer patients.

## Data Availability

Publicly available datasets were analyzed in this study. This data can be found here: http://www.cdc.gov/nchs/nhanes.

## Supplementary material

Supplementary Figure 1 Selection process for study cohorts.

Supplementary Figure 2 Association between AISI and all-cause mortality **(A)** and cardiovascular mortality **(B)** in participants with female cancer, adjusted for age, hypertension, diabetes, BMI, alcohol use, and smoking. The shaded areas represent the 95% CI.

Supplementary Table 1 Baseline characteristics of the study cohort.

Supplementary Table 2 Comparison of prognostic ability for all-cause and Cardiovascular mortality between AISI and SII.

Supplementary Table 3 HRs (95% CIs) for all-cause and cardiovascular mortality according to different types of AISI data in cancer patients from Dandong Central Hospital (2022–2023) among adult females.
