# Supplementary material for: Association of aggregate index of systemic inflammation with increased all-cause and cardiovascular mortality in female cancer patients
Source: Front Oncol. 2025 Apr 29;15:1552341. doi: 10.3389/fonc.2025.1552341 (PMC12069043; doi:10.3389/fonc.2025.1552341)
Supplement: Supplementary Figure 1 — Selection process for study cohorts. [file DataSheet1.docx]

**Appendix:**

**Supplementary Figure 1 Selection process for study cohorts.**

**Supplementary Figure 2 Association between AISI and all-cause mortality (A) and cardiovascular mortality (B) in participants with female cancer, adjusted for age, hypertension, diabetes, BMI, alcohol use, and smoking. The shaded areas represent the 95% CI.**

**Supplementary Table 1 Baseline characteristics of the study cohort.**

**Supplementary Table 2 Comparison of prognostic ability for all-cause and Cardiovascular mortality between AISI and SII.**

**Supplementary Table 3 HRs (95% CIs) for all-cause and cardiovascular mortality according to different types of AISI data in cancer patients from Dandong Central Hospital (2022–2023) among adult females.**

**Supplementary Figure 1 Selection process for study cohorts.**


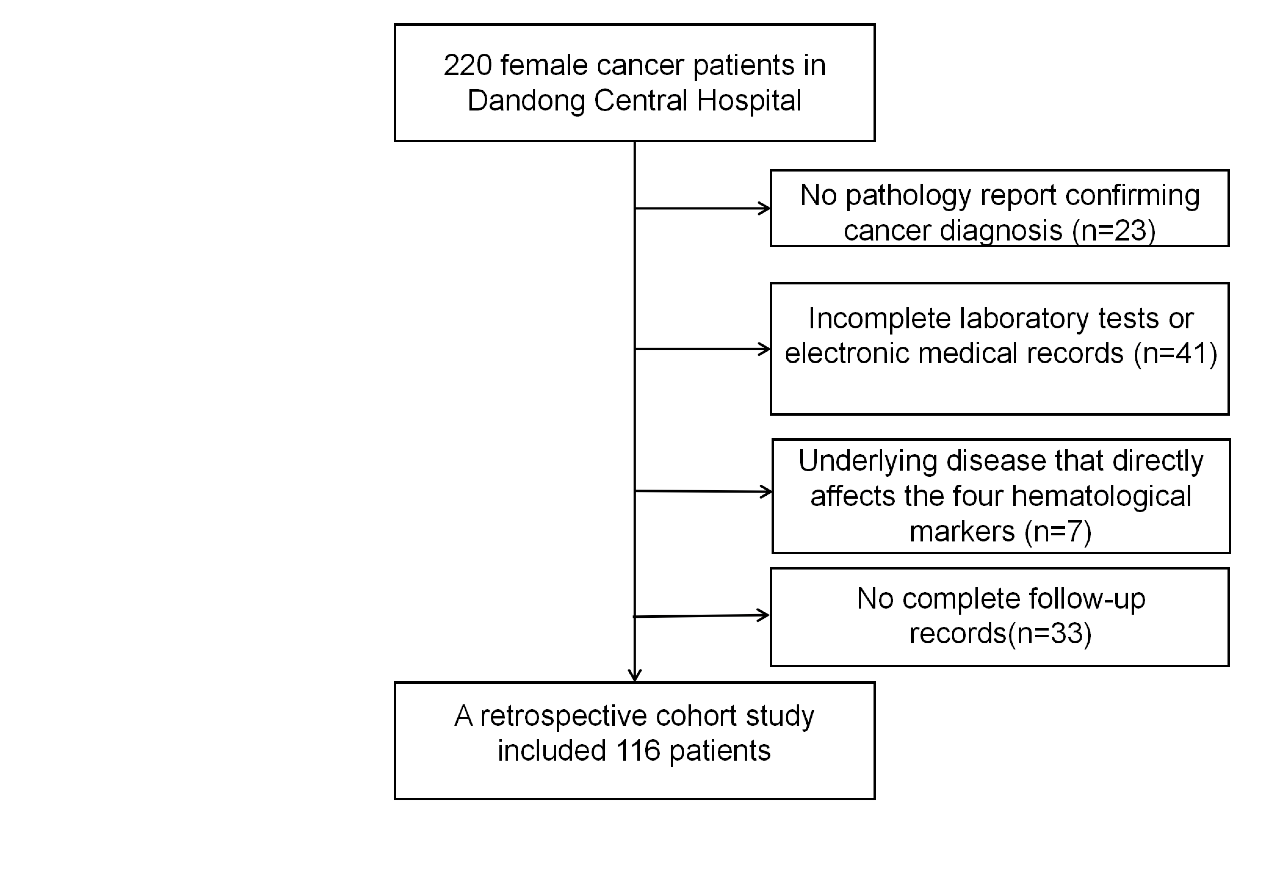


**Supplementary Figure 2 Association between AISI and all-cause mortality (A) and cardiovascular mortality (B) in participants with female cancer, adjusted for age, hypertension, diabetes, BMI, alcohol use, and smoking. The shaded areas represent the 95% CI.**


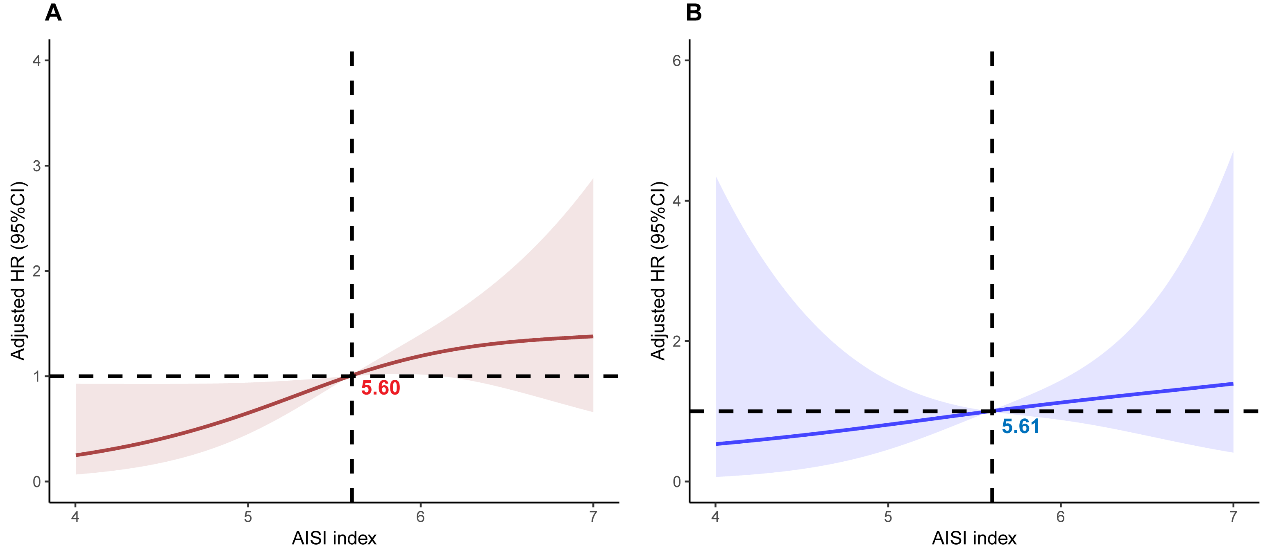


**Supplementary Table 1 Baseline characteristics of the study cohort.**

| **Study variables** | | **Total**  **(n=116)** | **Quartiles of AISI** | | | | **P value** |
| --- | --- | --- | --- | --- | --- | --- | --- |
|  |  |  | **Q1<4.99**  **(n = 29)** | **Q2：4.99-5.58**  **(n = 29)** | **Q3:5.58-6.34**  **(n = 29)** | **Q4>6.34**  **(n = 29)** |  |
| **Age,years** | | **64.22 ± 9.00** | **61.41 ± 10.66** | **65.66 ± 7.76** | **65.76 ± 8.32** | **64.07 ± 8.77** | **0.221** |
| **Smoking status, n (%)** | |  |  |  |  |  | **0.933** |
|  | **No** | **70 (60.34%)** | **18 (62.07%)** | **16 (55.17%)** | **18 (62.07%)** | **18 (62.07%)** |  |
|  | **Yes** | **46 (39.66%)** | **11 (37.93%)** | **13 (44.83%)** | **11 (37.93%)** | **11 (37.93%)** |  |
| **Alcohol use, n (%)** | |  |  |  |  |  | **0.458** |
|  | **No** | **31 (26.72%)** | **11 (37.93%)** | **7 (24.14%)** | **6 (20.69%)** | **7 (24.14%)** |  |
|  | **Yes** | **85 (73.28%)** | **18 (62.07%)** | **22 (75.86%)** | **23 (79.31%)** | **22 (75.86%)** |  |
| **Hypertension, n (%)** | | **48 (41.38%)** | **9 (31.03%)** | **15 (51.72%)** | **15 (51.72%)** | **9 (31.03%)** | **0.163** |
| **Diabetes mellitus, n (%)** | | **21 (18.10%)** | **9 (31.03%)** | **2 (6.90%)** | **5 (17.24%)** | **5 (17.24%)** | **0.124** |
| **BMI, kg/m2** | | **22.57 ± 2.93** | **22.14 ± 2.90** | **23.13 ± 2.82** | **22.87 ± 2.94** | **22.14 ± 3.09** | **0.467** |
| **Laboratory tests** | |  |  |  |  |  |  |
|  | **Neutrophil count, 109/L** | **4.74 ± 2.58** | **2.81 ± 1.26** | **3.47 ± 1.02** | **5.21 ± 2.10** | **7.46 ± 2.61** | **<0.001** |
|  | **Lymphocyte count, 109/L** | **1.74 ± 0.75** | **1.96 ± 0.89** | **1.88 ± 0.77** | **1.60 ± 0.72** | **1.52 ± 0.50** | **0.065** |
|  | **monocytes count, 109/L** | **0.49 ± 0.21** | **0.31 ± 0.14** | **0.53 ± 0.17** | **0.47 ± 0.15** | **0.67 ± 0.20** | **<0.001** |
|  | **Platelet count, 109/L** | **252.39 ± 93.48** | **192.41 ± 79.05** | **219.86 ± 69.15** | **256.07 ± 57.95** | **341.21 ± 92.17** | **<0.001** |

**Supplementary Table 2 Comparison of prognostic ability for all-cause and Cardiovascular mortality between AISI and SII.**

|  | **C-index** | **IDI** | **P value** | **NRI** | **P value** |
| --- | --- | --- | --- | --- | --- |
| **All-cause mortality** |  |  |  |  |  |
| **AISI** | **0.797 (0.780, 0.814)** | **Reference** |  | **Reference** |  |
| **SII** | **0.588 (0.564, 0.613)** | **-0.295(-0.339, -0.266)** | **<0.0001** | **-0.524(-0.587, -0.482)** | **<0.0001** |
| **Cardiovascular mortality** |  |  |  |  |  |
| **AISI** | **0.848 (0.817, 0.878)** | **Reference** |  | **Reference** |  |
| **SII** | **0.621 (0.569, 0.673)** | **-0.138(-0.222, -0.111)** | **<0.0001** | **-0.577(-0.663, -0.493)** | **<0.0001** |

**Abbreviations: NRI, net reclassification improvement; IDI, integrated discrimination improvement**

**Supplementary Table 3 HRs (95% CIs) for all-cause and cardiovascular mortality according to different types of AISI data in cancer patients from Dandong Central Hospital (2022–2023) among adult females.**

| **ALI** | | **Model1❊** |  |  | **Model2❊** |  |
| --- | --- | --- | --- | --- | --- | --- |
|  |  | **HR (95% CI)** | **p value** |  | **HR (95% CI)** | **p value** |
| **All-cause mortality** | | | | | | |
| **Continuous data** |  | **1.38(0.99,1.95)** | **0.0605** |  | **1.57(1.12,2.21)** | **0.0084** |
| **Quartiles** | **Q1** | **Reference** |  |  | **Reference** |  |
|  | **Q2** | **0.95(0.32,2.84)** | **0.9319** |  | **1.78(0.55,5.82)** | **0.3392** |
|  | **Q3** | **1.88(0.67,5.29)** | **0.2319** |  | **3.24(1.07,9.79)** | **0.0368** |
|  | **Q4** | **1.87(0.68,5.18)** | **0.2283** |  | **3.40(1.12,10.31)** | **0.0311** |
| **Cardiovascular mortality** | | | | | | |
| **Continuous data** |  | **1.41(0.81,2.46)** | **0.2235** |  | **1.34(0.75,2.39)** | **0.3226** |

**CI, Confidence Interval; HR, Hazard Ratio; AISI, The aggregate index of systemic inflammation.**

**❊Model 1, non-adjusted. Model 2, adjusted for age, hypertension, diabetes, alcohol use, smoking and BMI.**
